# Ovarian germ cell tumors with rhabdomyosarcomatous components and later development of growing teratoma syndrome: a case report

**DOI:** 10.1186/1752-1947-6-13

**Published:** 2012-01-16

**Authors:** Usama Al-Jumaily, Maysa Al-Hussaini, Fatenah Ajlouni, Abdulrahman Abulruz, Iyad Sultan

**Affiliations:** 1 Department of Pediatric Oncology, King Hussein Cancer Center, Queen Rania Al Abdullah St., Amman, 11941, Jordan; 2 Department of Pathology, King Hussein Cancer Center, Queen Rania Al Abdullah St., Amman, 11941, Jordan; 3 Department of Radiology, King Hussein Cancer Center, Queen Rania Al Abdullah St., Amman, 11941, Jordan

## Abstract

**Introduction:**

Development of a sarcomatous component in a germ cell tumor is an uncommon phenomenon. Most cases reported have a grim prognosis. Growing teratoma syndrome is also an uncommon phenomenon and occurs in approximately 2% to 7% of non seminomatous germ cell tumors and should be treated surgically.

**Case presentation:**

We report the case of a 12-year-old Asian girl with an ovarian mixed germ cell tumor containing a rhabdomyosarcomatous component. She was treated with a germ cell tumor chemotherapy regimen and rhabdomyosarcoma-specific chemotherapy. Towards the end of her treatment, she developed a retroperitoneal mass that was increasing in size. It was completely resected, revealing a mature teratoma, consistent with growing teratoma syndrome. She is still in complete remission approximately three years after presentation.

**Conclusion:**

The presence of rhabdomyosarcoma in a germ cell tumor should be treated by a combined chemotherapy regimen (for germ cell tumor and rhabdomyosarcoma). In addition, development of a mass during or after therapy with normal serum markers should raise the possibility of growing teratoma syndrome that should be treated surgically.

## Introduction

Development of a sarcomatous component (SC) in a germ cell tumor (GCT) is a rare phenomenon. Many histologic types of SC are described which can be present in the primary tumor or in the metastatic sites. The presence of sarcomas within GCTs is mostly encountered in testicular and mediastinal GCT while their presence in an ovarian GCT has rarely been reported. Meticulous histological examination is very important to determine the type and percentage of SC within a GCT as this affects prognosis. Growing teratoma syndrome (GTS) is also a rarely reported phenomenon. It was first described in 1982 [1] and occurs in approximately 2% to 7% of non-seminomatous germ cell tumors (NSGCT) [2,3]. It requires three criteria for definition: firstly, normalization of the previously elevated serum tumor markers (alpha fetoprotein (AFP) and beta human chorionic gonadotropins (B-HCG)); secondly, an increase in tumor size during and after chemotherapy given for NSGCT; and thirdly, the absence of any NSGCT component other than mature teratoma (MT) when the tumor is resected. Another characteristic finding of the GTS is the appearance of cysts within the growing masses which appear as an increase in mass size.

The patient's primary tumor histopathology, her response to chemotherapy, and development of rare GTS make this patient's case worth reporting.

### Case presentation

**Figure 1:**
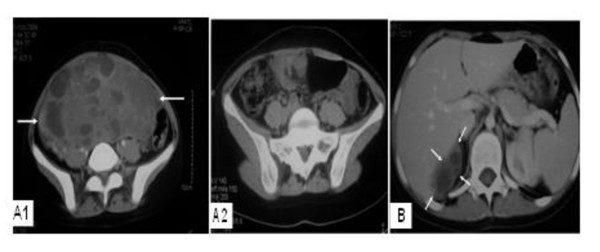
**Computerized Tomography findings before and during the course of treatment**. (**A1**) showing huge heterogeneous mass filling the upper pelviabdominal cavity (arrows), (**A2**) showing unremarkable pelvis after chemotherapy, (**B**) showing the appearance of a new mass situated between right liver lobe, right kidney, and adrenal gland (arrows).

**Figure 2:**
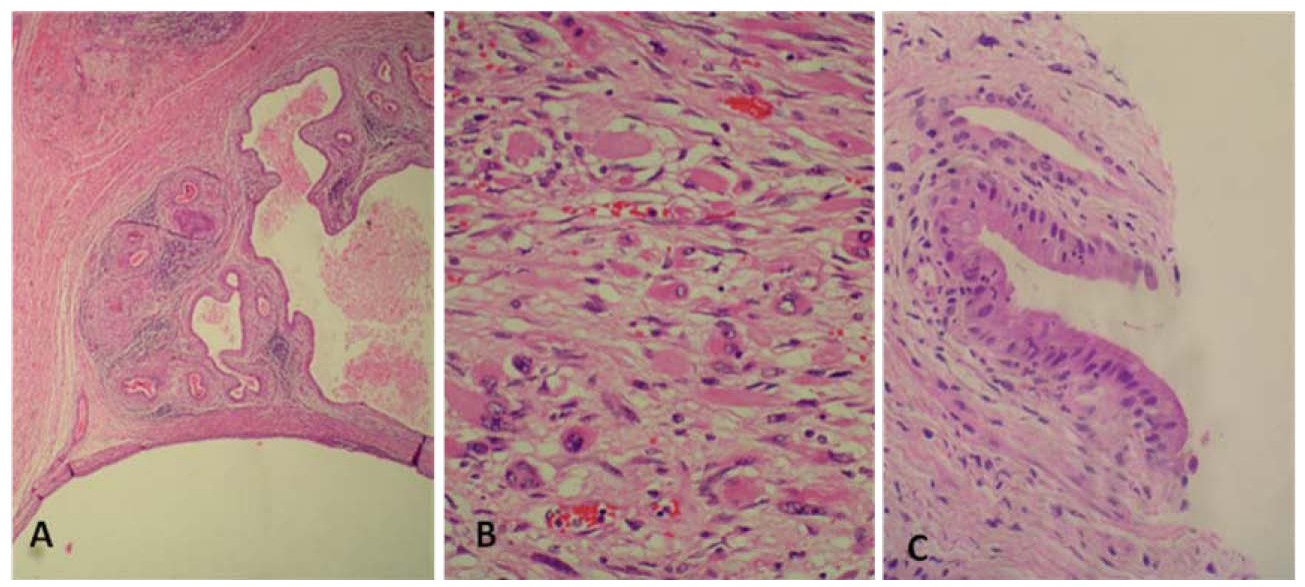
**Histological features of the primary ovarian mass and the recurrent new mass**. (**A**) low power magnification of the mature teratoma component, in which mature glands and stroma are seen (H&E X4), (**B**): higher power magnification of rhabdomyoblasts seen with abundant acidophilic cytoplasm and eccentric nuclei (H&E X 40) (inset show desmin positive staining of the rhabdomyoblasts), (**C**): mature glandular tissue is seen in the recurrent new mass, no immature tissue was identified (H&E X 40).

A 12-year- old Asian girl presented with lower abdominal pain and distension of two months duration. A non-tender abdominal mass was found by palpation. Past medical history was unremarkable. She had not had her menarche yet. Computerized tomography (CT) scan showed a huge, heterogeneously enhancing pelviabdominal mass with multiple cystic and necrotic areas, originating from the right ovary (Figure 1A). The results of alpha fetoprotein (AFP) and beta human chorionic gonadotropin (B-HCG) laboratory tests were both elevated (2793 ng/ml (normal < 10) and 27361 mIU/ml (normal < 2), respectively). She underwent laparotomy with removal of the mass, right salpingoopherectomy, partial omentectomy, iliac lymph nodes sampling, and ascetic fluid sampling. No intraoperative spillage was observed. Histopathology revealed non-germinomatous mixed germ cell composed of a mixture of yolk sac tumor (20%), mature teratoma (30%) (Figure 2A), embryonal carcinoma (40%), and choriocarcinoma (5%). In addition, an embryonal rhabdomyosarcomatous component (5%) (Figure 2B) was identified in the form of spindle and globoid rhabdomyoblasts staining positively with desmin and focally for myogenin. Iliac lymph nodes, omentum, and peritoneal excisional biopsies were free of tumor. Ascetic fluid cytology was also free of malignancy.

She was treated with eight courses of chemotherapy (Table 1). She was followed by serial AFP and B-HCG tests which both normalized after the third cycle of chemotherapy.

**Table 1:** Courses of chemotherapy used by the patient

Chemotherapy course number	Week	Chemotherapeutic agents
1	0	BEP

2	three	VAC

3	six	BEP

4	nine	BEP

5	12	BEP

6	15	BEP

7	18	BEP

8	21	VAC

9	24	VAC

10	27	VAC

BEP, Bleomycin, Etoposide, Cisplatin; VAC, Vincristine, Actinomycin, and Cyclophosphamide

Six months after presentation and while she was on chemotherapy a right hypochondrial mass was detected by physical examination. Imaging studies revealed a new complex multiloculated mass in the right suprarenal area (Figure 1B). Serum markers were normal. Computerized tomography-guided biopsy revealed a mature teratoma. Later there was a 30% increase in the size of the mass. She then underwent laparotomy with complete resection of the mass. Histopathology confirmed the presence of mature cystic teratoma (Figure 2C).

She is still in complete remission 32 months after presentation.

## Discussion

We describe an ovarian mixed GCT with rhabdomyosarcomatous components and elevated serum AFP and B-HCG in an adolescent girl. The development of SC in GCT is an uncommon phenomenon. Histologic types of SC reported in the literature are the following: rhabdomyosarcoma, high grade unclassified sarcoma, rhabdomyosarcoma admixed with high-grade unclassified sarcoma, angiosarcoma, and low-grade myxoid sarcoma [4-7]. The sarcomatous element can be present in the primary tumor or it can appear in the metastases [7]. The occurrence of sarcomas within GCTs has mostly been encountered in testicular and mediastinal GCT while their presence in an ovarian GCT has rarely been reported [7-10]. The sarcomatous component is of paramount importance because of its aggressive behavior, tendency for metastasis, and poor prognosis and thus may support the inclusion of sarcoma-oriented drugs for this particular group [8]. The proportion of SC within germ cell tumors appears to have influenced the prognosis (that is, the higher the percentage of SC within GCT the poorer the prognosis) [11]. So the importance of thorough sampling and meticulous histological examination for determining the type and extent of the malignant component should be emphasized.

The largest study was published by Malagon *et al. *They identified 46 cases of GCT with SC. Rhabdomyosarcomatous (RMS) components were found in 28 cases. Only two patients were in the pediatric age group. Details of treatment were not reviewed, however most patients were treated recently, suggesting the use of multimodality treatment. Nevertheless, the outcome of these patients appeared to be less than favorable [8].

Although the pathogenesis of the development of sarcomatous components in GCT has not been fully elucidated, origins proposed have included dedifferentiation or malignant transformation of certain mesenchymal components within teratomas, origin from primitive germ cells or transformation of the blastematous stroma in yolk sac tumor [7,8,12]. Upon review of the literature it is clear that most GCT with SC (whether in the primary tumor or in the metastases) have a teratoma component as well, which might support the first theory [12]. Noteworthy is the notion that the old terminology of the World Health Organization (WHO) classification of the presence of non-germ cell malignancies within germ cell neoplasms was 'teratoma with malignant transformation' or 'teratoma with malignant areas' [13].

GTS is a very rare phenomenon. Prognosis is favorable when surgery is radical [1,3]. Otherwise the outcome may be grim [14]. Our patient fulfilled the criteria of GTS and so was treated with radical resection only. Although GTS is mostly reported in adults, we have identified some pediatric cases (Table 2) [15-19].

**Table 2:** Review of pediatric cases with GTS

	Number of patients reported	Original tumor site	Age at diagnosis	Original pathology	Follow up
Kong DS et al [14]	six	intracranial	Two months 17 yr	4 mixed GCT, 2 IT	NA

Tangjitgamol S et al [15]	one	ovarian	five yr	IT	Developed another recurrence two years later and then underwent another surgery

Amsalem H. et al [16]	one	ovarian	12 yr	IT	NA

Nimkin K et al [17]	one	Ovarian	12 yr	IT	NA

Inaoka T et al [18]	one	Ovarian	five yr	IT	NA

GCT, germ cell tumors; IT, immature teratoma; NA, not available

The pathogenesis of the GTS is still unclear but two mechanisms have been considered: malignant cell differentiation into mature teratoma (MT) or a chemotherapy-induced destruction of the component other than mature teratoma (that is, the killing of malignant cells by chemotherapy with concomitant MT enlargement) [20]. As our patient had a rhabdomyosarcomatous component in the initial histology, we are more in favor of the second mechanism.

Successful pregnancy after development of GTS has been reported indicating the necessity of a fertility sparing surgical approach in the treatment of young female patients [21].

Many factors have been proposed that might predict the subsequent development of a GTS including the following: presence of MT in the primary NSGCT; no reduction in the size of metastases during chemotherapy; and the presence of MT in post chemotherapy residual masses [20].

Clinical complications associated with GTS were estimated as approximately 12% and generally related to organ compression [20]. Malignant transformation may occur as well, albeit at a much lower rate (3%) [20] and tends to be encountered more in adults. This includes transformation into malignant NSGCT, sarcoma, squamous cell carcinoma, adenocarcinoma, carcinoid tumor and primitive neuroectodermal tumor (PNET).

Alpha-interferon therapy has been reported as an option for treatment of recurrent mature teratoma although it was not confirmed in a cohort of patients [22]. New investigational therapy using selective cyclin dependent kinase CDK inhibitors suggests a new treatment for growing teratoma syndrome especially in those with unresected or recurrent GTS [23].

## Conclusions

We report a rare case of an ovarian mixed GCT associated with a rhabdomyosarcomatous component which was treated successfully using a combined regimen (for GCT and RMS). The case was also complicated by the occurrence of the GTS which was treated surgically.

## Consent

Written informed consent was obtained from the patient's next-of-kin for publication of this case report and any accompanying images. A copy of the written consent is available for review by the Editor-in-Chief of this journal.

## Competing interests

The authors declare that they have no competing interests.

## Authors' contributions

UA interpreted the patient data and was a major contributor to the writing of the manuscript. AA collected the clinical data. MAlh obtained and interpreted pathological studies. FAj obtained and interpreted radiological studies. UA and IS reviewed the literature. All authors read and approved the final manuscript.
